# A STAKEHOLDER-PARTNERED APPROACH TO INEQUITIES AFFECTING HOME HEALTH CARE WORKERS, OLDER ADULTS, AND CAREGIVERS

**DOI:** 10.1093/geroni/igad104.1802

**Published:** 2023-12-21

**Authors:** Olivia Okereke, Vivian Anable Eme, Keliane Totten, Joseph Locascio, Alison Simmons, Nancy Carpenter, Anthony Weiner

**Affiliations:** Massachusetts General Hospital/Harvard Medical School, Boston, Massachusetts, United States; Massachusetts General Hospital, Boston, Massachusetts, United States; Pear Associates, LLC, Wellesley, Massachusetts, United States; Biostatistics, Massachusetts General Hospital, Boston, Massachusetts, United States; Center for Community Health Education Research and Service (CCHERS), Boston, Massachusetts, United States; Center for Community Health Education Research and Service (CCHERS), Boston, Massachusetts, United States; Geriatric Psychiatry, Massachusetts General Hospital, Boston, Massachusetts, United States

## Abstract

Home health aides (HHAs) provide on-site support for homebound older adults with cognitive impairments, while lessening strain on familial caregivers. However, HHAs face structural challenges in work-related transportation. Massachusetts General Hospital (MGH) researchers partnered with community and corporate stakeholders to inform a study evaluating whether on-demand transportation for HHAs via Uber rides will: 1) Improve metrics among HHAs (total visits, total unique patients, hours/week and days/week worked, missed visit/no-show rates, work satisfaction); 2) Increase racial, ethnic, socioeconomic and geographic diversity of older adults with cognitive impairments receiving homecare. Community partner CCHERS (Center for Community Health Education Research Services) helps residents of public/publicly-assisted housing in high-need areas of Greater Boston gain training and entry-level employment in healthcare as HHAs. Through its collaborations with other groups (e.g., Mothers for Justice & Equality, HomeCare Aide Council, Mass HomeCare Alliance), CCHERS identified that lack of affordable, accessible, reliable transportation was among the most-cited barriers affecting HHAs and equitable homecare delivery. Corporate partner Uber Health, through work in preliminary social-impact case studies, identified the platform’s central ride-coordination approach as preferred by Black and Hispanic women in the 18-45y age group (~80% of the local HHA workforce). MGH researchers’ study goals and metrics were informed by input of partners. With its community-engaged approach, innovative partnerships, and focus on achieving equity objectives to benefit patients and homecare workers alike, this project has potential for strong local impacts and future implications for state and/or national policies to provide high-quality, equitable homecare by directly addressing employment transportation-related barriers.

